# Our Grandmothers’ Herb Garden

**Published:** 1914-06

**Authors:** 


### OUR GRANDMOTHERS' HERB GARDEN

### Old Fashioned Remedies and Their Uses.

**Colds.** A drink of hot Sage Tea on going to bed. Pennyroyal or Catnip can be used in place of Sage. Horehound, sweetened with Honey, to relieve a cough, or Garden Hyssop will answer. A poultice of Hops and Ryemeal applied to the neck or chest. Thoroughwort and Ginger mixed, to remove all relics of a cold. Cider and Molasses, steeped with a pod or two of Red Peppers, and drunk hot.

**Rheumatism.** Wormwood, or Wormwood and Peppermint mixed. Red Peppers in Cider Vinegar.

**Stitch in Side.** A plaster of Pitch and Apple.

**Neuralgia in the Thigh.** A poultice of cold raw Carrots, pound or grate till smooth and soft.

**Felons.** A poultice of the root of Wild Evening Primrose, grate or pound to pulp, boil in milk or water, thicken with cracker, and apply hot.

**Spring Medicine.** A few chips of Black Birch inner bark, a few sprigs of Hemlock or Spruce, a handful of Checkerberry Root, a small root of Horseradish, some roots of Wild Sarsaparilla, some chips of White Pine, a small amount of the bark of Sassafras root, a handful of roots and leaves of Pipsissewa, the same of Pear Leaf Wintergreen and of Lettuce Liverwort. Brew in water, strain into jar, add water, sweeten with Molasses or Brown Sugar to taste. When cool, add a cup of Hop Yeast. Keep in warm place until fermented, then bottle and keep in cool place.

**Cuts, Strains and Bruises.** Fill pint bottle half full of buds of Balm of Gilead gathered in April, then fill bottle with New England Rum. Cork tightly. Collect the cotton bloom from Ament, and use as lint for cuts and bruises.

**Black and Blue Spots.** The root of Solomon's Seal, fresh and green.

**Jaundice.** Yellowdock and Barberry Bark, steeped in water and drunk before meals. Black, or Rum, Cherries preserved in spirits.

**Ticdouloureaux.** A hot boiled Potato poultice, applied to the affected parts.

**Sunburn.** Green plantain leaves.

**Loss of Sleep.** A pillow of Hops, carefully dried so as not to lose the pollen, or a pillow of Life Everlasting Flowers gathered before frost.

**Inflamed Eyes.** Boiling water poured on the Pitch of Elder.

**Earache.** The core of a roasted Onion, applied hot.

**Burns and Cracked Hands.** Equal parts of Houseleek and green bark of Elder, simmered in milk and water till soft, strain to remove all dregs, add a part of fresh churned unsalted butter, a bit of bee's wax, simmer again until smooth, then place in a big mouthed bottle and set away for future use.

**Nausea.** Spearmint Tea, sweetened, and drunk hot.

Am. Pract., Jan., 1914.

